# Supplementary material for: Content-rich biological network constructed by mining PubMed abstracts
Source: BMC Bioinformatics. 2004 Oct 8;5:147. doi: 10.1186/1471-2105-5-147 (PMC528731; doi:10.1186/1471-2105-5-147)
Supplement: Additional File 2 — The original results of the above study (non-essential files are deleted to keep the file size under the limit set by BMC bioinformatics). [file 1471-2105-5-147-S2.bz2 › chilibotAdditionalFile2/dip50/263ID7973658E1060/html/IL2RG_JAK3.html]

 


 **IL2RG** and **JAK3** 
  
Found 74 abstracts in PubMed, retrieved 50.  
 

 What does Google say? 
 PDF only 
| .edu only 

---

**Interactive relationship** (e.g. stimulation, inhibition, etc)

**Neutral relationship**- As a result of this interaction, p12 I increases signal transducers and activators of transcription 5 STAT5 DNA binding and transcriptional activity and this effect depends on the presence of both IL 2R beta and gamma c  [ **IL2RG** ]  chains and  **Jak3** .  Ref: 11468184 Blood, 2001
- Therefore, fusion proteins were constructed in which all or part of the cytoplasmic domain of gamma c  [ **IL2RG** ]  was replaced by  **Jak3** .  Ref: 9050873 Proc Natl Acad Sci U S A, 1997
- Heterotrimeric IL 2R alpha, beta, gamma c  [ **IL2RG** ]  chains activates Jak1 and  **Jak3** .  Ref: 9462834 FEBS Lett, 1998
- It is generally held that the IL 2R is activated by the trans activity of the protein tyrosine kinases PTKs Jak1 and  **Jak3**  associated with beta c and gamma c  [ **IL2RG** ]  respectively.  Ref: 10889465 Cell Signal, 2000
- Genetic and molecular studies have demonstrated that  **Jak3**  is essential for mitogenic signaling by the gamma c  [ **IL2RG** ]  chain.  Ref: 9050873 Proc Natl Acad Sci U S A, 1997
- Such signals are likely relayed inside the cell by the tyrosine kinase  **Jak3** , which associates with gamma c  [ **IL2RG** ] .  Ref: 9850851 Immunol Rev, 1998
- The  **JAK3**  tyrosine kinase is constitutively associated with the gamma c  [ **IL2RG** ]  and is necessary for signaling through the gamma c  [ **IL2RG** ]  containing receptors.  Ref: 9801259 Semin Hematol, 1998
- However, tyrosine phosphorylation of JAK 3  [ **JAK3** ] , which associates with gamma c  [ **IL2RG** ] , is not induced by SCF in these cells.  Ref: 9190938 J Immunol, 1997
- Here we show that in normal lung fibroblasts IL 4 and IL 13 induce the expression of the gamma c  [ **IL2RG** ]  chain and its association with Janus kinase 3  [ **JAK3** ] , while lung myofibroblasts constitutively express a gamma c  [ **IL2RG** ]  chain displaying a limited association with  **JAK3** .  Ref: 12207328 Eur J Immunol, 2002
- Taken together, these results definitively demonstrate that IL 21 mediated signaling requires the gamma c  [ **IL2RG** ]  chain, and indicate that  **JAK3**  is an essential transducer of gamma c  [ **IL2RG** ]  dependent survival and or mitogenic signals induced by this cytokine.  Ref: 12093291 Biochemistry, 2002
- Defects of the common gamma chain subunit of the cytokine receptors gamma c  [ **IL2RG** ]  or of  **Jak3** , a tyrosine kinase required for gamma c  [ **IL2RG** ]  signal transduction, result in T B severe combined immunodeficiency SCID.  Ref: 9446656 Blood, 1998
- The Janus family tyrosine kinase 3  **Jak3**  is known to be associated with gamma c  [ **IL2RG** ] , and the reported phenotypes of gamma c  [ **IL2RG** ]  deficient gamma c  [ **IL2RG** ]  and  **Jak3**  deficient  **Jak3**  mice are similar, indicating that  **Jak3**  is an essential transducer of gamma c  [ **IL2RG** ]  dependent signals.  Ref: 10653847 Int Immunol, 2000
- The interleukin 2 receptor IL 2R generates proliferative signals in T lymphocytes by ligand induced heterodimerization of two chains, IL 2Rbeta and gamma c  [ **IL2RG** ] , which associate with the tyrosine kinases Jak1 and  **Jak3** , respectively.  Ref: 9050873 Proc Natl Acad Sci U S A, 1997
- Furthermore, IL 7R gamma c  [ **IL2RG** ]  chain association correlates with the expression of JAK 3  [ **JAK3** ]  in T cells, but not in transfected COS 7 cells.  Ref: 9190922 J Immunol, 1997
- Nonreceptor tyrosine kinase  **Jak3** , which binds to intracellular domain of interleukin 2 receptor gamma  [ **IL2RG** ]  subunit IL 2Rgamma, plays an important role in IL 2 function.  Ref: 12136181 Sheng Wu Hua Xue Yu Sheng Wu Wu Li Xue Bao Shanghai < MedlineTA>Sheng Wu Hua Xue Yu Sheng Wu Wu Li Xue Bao Shanghai , 1997
- Janus kinase 3  [ **JAK3** ]   **Jak3**  is essential for common cytokine receptor gamma chain gamma c  [ **IL2RG** ]  dependent signaling comparative analysis of gamma c  [ **IL2RG** ] ,  **Jak3** , and gamma c  [ **IL2RG** ]  and  **Jak3**  double deficient mice.  Ref: 10653847 Int Immunol, 2000
- The Jak family tyrosine kinase,  **Jak3** , is involved in signaling through cytokine receptors utilizing the common gamma chain gamma c  [ **IL2RG** ] .  Ref: 9366394 J Immunol, 1997
- Retroviral mediated transduction of wild type gamma c  [ **IL2RG** ]  into XSCID  [ **IL2RG** ]  JT cells restored function to the IL 21R, as shown by IL 21 induced tyrosine phosphorylation of JAK1 and  **JAK3** , and downstream activation of STAT5, in JT gamma c  [ **IL2RG** ]  cells as well as BaF3 IL 21R alpha and primary splenic B cells.  Ref: 12093291 Biochemistry, 2002
- Janus kinase 3  [ **JAK3** ]   **JAK3**  is a cytoplasmic tyrosine kinase required for T cell development and activated by cytokines that utilize the interleukin 2 IL 2 receptor common gamma chain gamma c  [ **IL2RG** ] .  Ref: 10380915 J Leukoc Biol, 1999
- No significant differences between cell lines were observed in IL 2 receptor chain alpha, beta, or gamma c  [ **IL2RG** ]  expression, in IL 2 mediated proliferation, or in IL 2 induced phosphorylated forms of Stat3, Stat5, Jak1, or  **Jak3** .  Ref: 10516077 J Virol, 1999
- **JAK3**  is involved in the signal transduction pathways of the IL 2, IL 4, IL7, IL 9, and IL 15 receptors by association with their common gamma chain gamma c  [ **IL2RG** ] .  Ref: 9168059 Exp Hematol, 1997
- These results indicate that  **Jak3**  is absolutely essential for gamma c  [ **IL2RG** ]  dependent T cell and B cell development, and for gamma c  [ **IL2RG** ]  dependent prevention of thymocyte apoptosis.  Ref: 10653847 Int Immunol, 2000
- In XSCID  [ **IL2RG** ]  and SCID resulting from mutations in  **JAK3** , which encodes a Janus family tyrosine kinase that couples to gamma c  [ **IL2RG** ]  and is required for gamma c  [ **IL2RG** ]  dependent signalling, T and natural killer NK cells are decreased but B cell numbers are normal T B NK SCID.  Ref: 9843216 Nat Genet, 1998
- Evidence from peripheral T cells indicates that survival signals arising from the intermediate affinity IL 2R lacking CD25 do not require the activation of Janus kinase 3  [ **JAK3** ]   **Jak3**  but do require the presence of the membrane proximal region of the gamma C  [ **IL2RG** ]  chain.  Ref: 12121224 Immunol Cell Biol, 2002
- Stimulation through the gamma c  [ **IL2RG** ]  related cytokine receptors restores  **JAK3**  expression and activation and rescues CD4 mediated T cell unresponsiveness.  Ref: 9637477 J Immunol, 1998

**Non-interactive relationship** (e.g. studied together, co-existance, homology, etc.)

- In contrast, alpha beta iIEL can be detected in IL 7 gene deleted animals, but not in gamma c  [ **IL2RG** ] , or in JAK 3  [ **JAK3** ]  deficient mice suggesting that alternative cytokines may be involved in development of iIEL alpha beta T cells, but not necessarily for gamma delta T cells.  Ref: 9505193 Int Rev Immunol, 1998
- A  **Jak3**  independent signalling pathway involving p56 lck, generating anti apoptotic signals, can be observed and requires the PROX domain of gamma c  [ **IL2RG** ] .  Ref: 10889465 Cell Signal, 2000
- Chimeric gamma c  [ **IL2RG** ]  chains containing only  **Jak3**  in the cytoplasmic domain failed to mediate proliferation of CTLL 2 cells, but addition of a conserved membrane proximal PROX domain of gamma c  [ **IL2RG** ]  in tandem with  **Jak3**  fully reconstituted gamma c  [ **IL2RG** ]  function.  Ref: 9050873 Proc Natl Acad Sci U S A, 1997
- Three critical events have been identified in the generation of the IL 2R signal for cell cycle progression, including heterodimerization of the cytoplasmic domains of the IL 2R beta and gamma c  [ **IL2RG** ]  chains, activation of the tyrosine kinase  **Jak3** , and phosphorylation of tyrosine residues on the IL 2R beta chain.  Ref: 9755337 Adv Immunol, 1998
- Defects in the gamma c  [ **IL2RG** ]  or  **Jak3**  result in T B severe combined immunodeficiency SCID.  Ref: 10573019 Eur J Hum Genet< MedlineTA>Eur J Hum Genet, 1998
- Immunoprecipitation and blotting of the LUst cell line with specific secondary antibodies demonstrated that the 140 kd phosphoprotein was the interleukin 4 receptor, the 130 kd phosphoprotein was Janus kinase 1, the 116 kd phosphoprotein was Janus kinase 3  [ **JAK3** ] , and the 65 kd phosphoprotein was the interleukin 2 receptor gamma chain  [ **IL2RG** ] .  Ref: 10612755 J Thorac Cardiovasc Surg, 2000
- Nevertheless, certain differences have been suggested related to the range of actions of gamma c  [ **IL2RG** ]  and  **Jak3** .  Ref: 10653847 Int Immunol, 2000
- In non activated monocytes, neither the gamma c  [ **IL2RG** ]  nor its associated signal transducing molecule,  **Jak3** , is expressed.  Ref: 9144438 Biochem Biophys Res Commun, 1997
- Together, these data demonstrate that the IL 4R alpha cytoplasmic domain is sufficient to activate JAK 1 and STAT6 and to induce expression of IL 4 target genes, thus identifying a mechanism by which IL 4 signaling can proceed in the absence of JAK 3  [ **JAK3** ]  and gamma c  [ **IL2RG** ] .  Ref: 9190938 J Immunol, 1997
- Analyses of peripheral T cells in  **Jak3**  and gamma c  [ **IL2RG** ]  mice have also revealed a number of abnormalities.  Ref: 9620658 J Leukoc Biol, 1998
- **Jak3**  and gamma c  [ **IL2RG** ]  deficient mice have pleiotropic defects that can be attributed to their inability to respond to multiple specific cytokines.  Ref: 9620658 J Leukoc Biol, 1998
- In the disease, gamma c  [ **IL2RG** ]  is expressed, but its binding to  **Jak3**  is reduced.  Ref: 12126929 Mol Genet Metab, 2002
- We show that, similar to gamma c  [ **IL2RG** ]  deficient mice, T cells from  **Jak3**  deficient mice have an increased susceptibility to apoptosis.  Ref: 9366394 J Immunol, 1997
- The presence of NK function in ADA deficient, autosomal recessive, and unknown type SCIDs, and low NK function in a majority of gamma c  [ **IL2RG** ]  and  **Jak3**  SCIDs indicates that some molecular lesions affect T, B, and NK cells gamma c  [ **IL2RG** ]  and  **Jak3** , others primarily T cells ADA deficiency, and others just T and B cells.  Ref: 9063412 J Pediatr, 1997
- Although the mast cells in gamma c  [ **IL2RG** ]  and  **Jak3**  mice were morphologically indistinguishable from those in wild type mice, the number of peritoneal mast cells was decreased in gamma c  [ **IL2RG** ]  and  **Jak3**  mice as compared with that in wild type mice.  Ref: 10979963 Blood, 2000
- **Jak3** , a member of the Janus tyrosine kinase family is an intracellular kinase functionally coupled to cytokine receptors that share a common gamma chain gamma c  [ **IL2RG** ] .  Ref: 10573019 Eur J Hum Genet< MedlineTA>Eur J Hum Genet, 2000
- In addition,  **Jak3**  and gamma c  [ **IL2RG** ]  T cells are more prone to undergo apoptosis than wild type T cells.  Ref: 9620658 J Leukoc Biol, 1998
- Overall, many of the lymphoid defects of  **Jak3**  and gamma c  [ **IL2RG** ]  deficient mice can be accounted for by the lack of IL 7R and IL 2R signaling.  Ref: 9620658 J Leukoc Biol, 1998
- With the exception that T and B cells in  **Jak3**  mice express high levels of gamma c  [ **IL2RG** ] , the defects in thymocyte and peripheral T cell and B cell development are indistinguishable among gamma c  [ **IL2RG** ] ,  **Jak3**  and gamma c  [ **IL2RG** ]   **Jak3**  mice.  Ref: 10653847 Int Immunol, 2000
- While extensive molecular and biochemical analysis of these patients has been instrumental in understanding better the biological properties of the gamma c  [ **IL2RG** ]  and  **JAK3**  protein, an unexpected phenotypic heterogeneity of gamma c  [ **IL2RG** ]  and  **JAK3**  deficiency has emerged, indicating the need for appropriate and extensive investigations even in patients with atypical presentations.  Ref: 11213805 Immunol Rev, 2000
- Interleukin 13 is a potent activator of  **JAK3**  and STAT6 in cells expressing interleukin 2 receptor gamma  [ **IL2RG** ]  and interleukin 4 receptor alpha.  Ref: 8920992 Biochem J, 1996
- In addition, IL 4Ralpha, gamma c  [ **IL2RG** ] , and  **Jak3** , but not IL 2Rbeta or IL 7Ralpha, were expressed in BMMCs.  Ref: 10979963 Blood, 2000
- Role of common cytokine receptor gamma chain gamma c  [ **IL2RG** ]  and  **Jak3**  dependent signaling in the proliferation and survival of murine mast cells.  Ref: 10979963 Blood, 2000
- Specifically,  **Jak3**  and gamma c  [ **IL2RG** ]  deficient mice have small thymuses revealing a defect in T cell development, and in addition, have functionally unresponsive peripheral T cells with an activated memory cell phenotype.  Ref: 9620658 J Leukoc Biol, 1998
- Interleukin 2, interleukin 4, and interleukin 7 all utilize the common gamma gamma c  [ **IL2RG** ]  receptor component at the cell surface of lymphocytes and the  **Jak3**  kinase molecule to transduce signals inside the cell.  Ref: 9372081 Curr Opin Hematol, 1996
- Interestingly, although Bcl 2 induction was previously suggested to be  **Jak3**  independent, IL 7 cannot induce Bcl 2 expression in CD4 single positive SP thymocytes in either gamma c  [ **IL2RG** ]  or  **Jak3**  mice nor can IL 7 rescue CD4 SP thymocytes from dexamethasone induced cell death in gamma c  [ **IL2RG** ]  or  **Jak3**  mice.  Ref: 10653847 Int Immunol, 2000
- These data underline the pivotal role of the gamma c  [ **IL2RG** ]  chain, CD40 CD40L,  **JAK3**  and IL 13 in the inflammatory like activation of lung myofibroblasts, the cell type restraint effects of IL 13 on these cells, and the potential usefulness of  **JAK3**  inhibitors in the treatment of asthma.  Ref: 12207328 Eur J Immunol, 2002
- However, in EDgamma 16 cells, a gamma c  [ **IL2RG** ]  transfected ED40515 cell line, IL 21 binds to the IL 21R and can activate Janus kinase JAK 1,  **JAK3** , STAT1, and STAT3.  Ref: 11418623 J Immunol, 2001
- IL 7 signal transduction, involving gamma c  [ **IL2RG** ]  and  **Jak3** , is required for successful recombination at the TCR gamma locus.  Ref: 11404375 J Leukoc Biol, 2001
- The regulatory roles of the common cytokine receptor gamma chain gamma c  [ **IL2RG** ]  and  **Jak3**  dependent signaling in the proliferation and survival of mast cells were determined using gamma c  [ **IL2RG** ]  deficient gamma c  [ **IL2RG** ]  and  **Jak3**  deficient  **Jak3**  mice.  Ref: 10979963 Blood, 2000
- Defects of the common gamma chain gamma c  [ **IL2RG** ]  or of the  **JAK3**  protein in humans have been shown to result in a severe combined immune deficiency SCID, with a profound defect in T and natural killer NK cell development.  Ref: 11213805 Immunol Rev, 2000
- Both gamma c  [ **IL2RG** ]  and  **Jak3**  kinase deficient animals display a phenotype similar to interleukin 7 deficient animals in terms of lymphoid development.  Ref: 9372081 Curr Opin Hematol, 1996
- The thymic defect in these mutant mice strongly resembles that seen in IL 7 and IL 7 receptor knockout mice, suggesting that the lack of IL 7 receptor signaling accounts for this defect in  **Jak3**  and gamma c  [ **IL2RG** ]  mice.  Ref: 9620658 J Leukoc Biol, 1998
- T cells with wild type gamma c  [ **IL2RG** ]  and  **JAK3**  proteins have a profound selective advantage over cells that contain mutant proteins.  Ref: 9801259 Semin Hematol, 1998
- Moreover, mutations in IL 2Ralpha, gamma c  [ **IL2RG** ]  and  **Jak3**  have been found in patients with severe combined immunodeficiency.  Ref: 9620644 Cytokine Growth Factor Rev, 1997
- These features resemble those observed in gamma c  [ **IL2RG** ]  y and in  **Jak3**  mice, in which a population of activated, anergic T cells predominantly CD4 also develops with age.  Ref: 9446656 Blood, 1998
- The common gamma chain gamma c  [ **IL2RG** ]  is a required signaling component of the IL 21 receptor and supports IL 21 induced cell proliferation via  **JAK3** .  Ref: 12093291 Biochemistry, 2002
- These results indicate that gamma c  [ **IL2RG** ]  and  **Jak3**  dependent signaling is essential for IL 4 and IL 9 induced proliferation and survival of murine mast cells, that the effects of IL 4 are mediated by type I IL 4R.  Ref: 10979963 Blood, 2000
- IL 4 phosphorylates the 65 kd isoform of Stat6 in mast cells in a gamma c  [ **IL2RG** ]  and  **Jak3**  dependent manner.  Ref: 10979963 Blood, 2000
- Moreover, we demonstrate a functional requirement for both gamma c  [ **IL2RG** ]  and the gamma c  [ **IL2RG** ]  associated Janus family tyrosine kinase 3  **JAK3**  in IL 21 induced proliferation of pro B lymphoid cells engineered to express human IL 21R alpha BaF3 IL 21R alpha.  Ref: 12093291 Biochemistry, 2002
- Forty nine had X linked SCID with mutations of common cytokine receptor gamma chain gamma c  [ **IL2RG** ] , 16 had adenosine deaminase ADA deficiency, 8 had Janus kinase 3  [ **JAK3** ]   **Jak3**  deficiency, 21 had unknown autosomal recessive mutations, 1 had reticular dysgenesis, 1 had cartilage hair hypoplasia, and 12 all boys had SCID of undetermined type.  Ref: 9063412 J Pediatr, 1997
- Defects of the gamma c  [ **IL2RG** ]   **JAK3**  signaling pathway as a model.  Ref: 11213805 Immunol Rev, 2000
- gamma c  [ **IL2RG** ]  or  **Jak3**  deficiency resulted in the most B cells and fewest natural killer NK cells.  Ref: 9063412 J Pediatr, 1997
- However, the effects of IL 4 and IL 9 were absent in BMMCs from gamma c  [ **IL2RG** ]  and  **Jak3**  mice.  Ref: 10979963 Blood, 2000
- In the latter cells, without exogenous cytokines, gamma c  [ **IL2RG** ]   **JAK3**  controls, through autocrine loops, tyrosine kinase 2 phosphorylation and the balance between functional and decoy high affinity receptors.  Ref: 12207328 Eur J Immunol, 2002
- To clarify whether gamma c  [ **IL2RG** ]  dependent cytokines can partially transduce their signals without  **Jak3** , we compared lymphocyte development in gamma c  [ **IL2RG** ] ,  **Jak3** , and gamma c  [ **IL2RG** ]  and  **Jak3**  double deficient gamma c  [ **IL2RG** ]   **Jak3**  mice in the same genetic background.  Ref: 10653847 Int Immunol, 2000
- Furthermore, IL 4 phosphorylated the 65 kd isoform of Stat6 in BMMCs from wild type mice but not from gamma c  [ **IL2RG** ]  and  **Jak3**  mice.  Ref: 10979963 Blood, 2000
- The discovery of these signaling pathways has led to important new insights into their role in lymphocyte maturation, as it has emerged that mutations in the genes encoding both gamma c  [ **IL2RG** ]  and  **JAK3**  result in similar forms of severe combined immunodeficiency SCID.  Ref: 8864127 J Leukoc Biol, 1996
- The shared common gamma chain gamma c  [ **IL2RG** ]  of IL 2R and its associated Janus kinase,  **JAK3** , are indispensable for normal T cell function.  Ref: 9637477 J Immunol, 1998
- because it is also the only molecule known to associate with gamma c  [ **IL2RG** ] , we speculated that  **Jak3**  might be sufficient for signaling by this chain.  Ref: 9050873 Proc Natl Acad Sci U S A, 1997
